# Association between MDROs colonization and subsequent infections in ICU patients: a retrospective cohort study

**DOI:** 10.3389/fpubh.2026.1863334

**Published:** 2026-07-13

**Authors:** Shanbi Cheng, Dejun Tong, Qianqian Zhao, Bing Xiao, Yudong Zhang, Yibo Gong

**Affiliations:** 1 Department of Hospital Infection Control, The Second Xiangya Hospital Central South University, Changsha, Hunan, China; 2 Department of Emergency, The Second Xiangya Hospital, Central South University, Changsha, Hunan, China; 3 Guilin Hospital of the Second Xiangya Hospital, Central South University, Changsha, Hunan, China; 4 Simulation Medicine Center, The Second Xiangya Hospital, Central South University, Changsha, Hunan, China; 5 Department of Cardiac Surgery, The Second Xiangya Hospital Central South University, Changsha, Hunan, China

**Keywords:** colonization, ICU, infection, multidrug-resistant organisms, risk factors

## Abstract

**Objective:**

To investigate the association between multidrug-resistant organisms (MDROs) colonization and subsequent infection in intensive care unit (ICU) patients.

**Methods:**

A retrospective analysis was conducted on 388 ICU patients admitted to the Second Xiangya Hospital between January 2023 and December 2025. Patients were divided into infection (*n* = 47) and non-infection (*n* = 341) groups according to whether they developed an MDROs infection within 28 days of ICU admission. Clinical data were collected, and univariate and multivariate logistic regression analyses were performed to identify the factors associated with MDROs infection. A nomogram model was constructed.

**Results:**

Among the 388 ICU patients, 99 (25.52, 95%CI: 21.44–30.08%) were colonized by MDROs. A total of 108 MDROs isolates were detected, including 29 strains of ESBL-producing *Escherichia coli* (ESBL-EC, 26.61%), 54 strains of ESBL-producing *Klebsiella pneumoniae* (ESBL-KP, 49.54%), 16 strains of methicillin-resistant *Staphylococcus aureus* (MRSA, 14.68%), and 9 strains of methicillin-resistant *Staphylococcus epidermidis* (MRSE, 9.17%). Logistic regression analysis showed that diabetes (OR = 2.523, 95% CI: 1.095–5.814), MDROs colonization (OR = 5.155, 95% CI: 2.302–11.544), APACHE II score (OR = 2.043, 95% CI: 1.597–2.614), and albumin level (OR = 0.778, 95% CI: 0.679–0.893) were independent factors associated with MDROs infection in ICU patients. When these variables were incorporated into a logistic regression nomogram, the AUC values for the training and validation sets were 0.894 (95% CI: 0.832–0.957) and 0.896 (95% CI: 0.828–0.964), respectively.

**Conclusion:**

Diabetes, MDROs colonization, high APACHE II scores, and low albumin levels are factors associated with MDROs infection in ICU patients. The nomogram constructed based on these factors may facilitate the early identification of patients at high risk for MDROs infection and provide a basis for individualized treatment strategies.

## Introduction

Multidrug-resistant organisms (MDROs) primarily refer to bacteria that are simultaneously resistant to three or more classes of clinically used antimicrobial agents simultaneously (1). The detection rate of MDROs has increased annually in recent years. Infections caused by MDROs complicate the clinical treatment and nursing care of critically ill patients in intensive care units (ICUs). These infections are associated with various complications, poor patient outcomes, and increased burden on families and society (2, 3).

Colonization refers to the presence and proliferation of bacteria in a specific part of the human body without causing tissue damage, triggering a host immune response, or producing clinical symptoms. Patients in ICUs typically have severe underlying diseases and acute physiological dysfunction and are often in an immunosuppressed state. In addition, the frequent use of invasive procedures in ICUs makes these units high-risk areas for colonization by drug-resistant bacteria (4). Bacterial colonization is an important risk factor for infection; MDROs infections may occur when host immunity is compromised or the microbiota is disrupted (5). Therefore, active screening for MDROs colonization may contribute to the prevention of MDROs infection (6).

A review investigating the risk of infection associated with colonization by multidrug-resistant gram-negative bacteria reported that the pooled incidence of infection following colonization with ESBL-E and CRE was 22% (7). However, current research on the association between MDROs colonization and subsequent infections remains limited. Therefore, this study retrospectively analyzed the clinical data of ICU patients at the Second Xiangya Hospital to identify independent factors associated with MDROs infection and construct a nomogram prediction model. In addition, the association between MDROs colonization and subsequent infections was further evaluated using correlation analysis. The findings of this study may help clinicians identify ICU patients at a high risk of MDROs infection at an early stage and optimize treatment strategies.

## Materials and methods

### Baseline data

A retrospective analysis was conducted on 388 ICU patients admitted to the hospital between January 2023 and December 2025. Patients were divided into infection (*n* = 47) and non-infection (*n* = 341) groups according to whether they developed an MDROs infection within 28 days. Multidrug-resistant bacteria were defined as bacteria that were not susceptible to three or more classes of antimicrobial agents (8).

The inclusion criteria were as follows: (1) age > 18 years, (2) ICU stay ≥ 48 h, and (3) proactive MDROs screening performed within 48 h of ICU admission. The exclusion criteria were as follows: (1) presence of MDROs infection prior to ICU admission; (2) refusal of sampling by the patient or family members, or inability to cooperate due to severe illness; and (3) incomplete clinical data. This study was approved by the Ethics Committee of the Second Xiangya Hospital.

### Testing methods

#### Sampling and culture

① Nasal swab: A cotton swab moistened with sterile saline was gently inserted 2–3 cm into one nostril and rotated three times along the nasal wall before removal. Using the same swab and procedure, the other nostril was sampled in the same manner (for patients receiving enteral feeding, sampling from only one nostril was permitted). ② Anal swab: A cotton swab moistened with sterile saline was inserted 3–4 cm into the anus and gently rotated three times to collect the specimen.

All specimens were inoculated within 2 h after collection onto blood agar plates, MacConkey agar plates (with carbapenem antibiotic susceptibility cards attached), chocolate agar plates, and *Staphylococcus aureus* screening plates (Hefei Tianda Diagnostic Reagent Co., Ltd.) for bacterial culture.

#### Microbial identification and antimicrobial susceptibility testing

Suspected colonies were identified using matrix-assisted laser desorption/ionization time-of-flight mass spectrometry (MALDI-TOF MS) (Bruker, United States). Antimicrobial susceptibility testing was performed using the VITEK-2 Compact automated bacterial identification and susceptibility testing system. All operational procedures and interpretations of susceptibility results were conducted in accordance with the standards of the Clinical and Laboratory Standards Institute (CLSI). *Escherichia coli* ATCC 25922 and *Staphylococcus aureus* ATCC 29213 were used as quality-control strains.

MDROs primarily refer to bacteria that are simultaneously resistant to three or more classes of clinically used antimicrobial agents simultaneously. The MDROs monitored in this study included extended-spectrum β-lactamase-producing *Escherichia coli* (ESBL-EC), extended-spectrum β-lactamase-producing *Klebsiella pneumoniae* (ESBL-KP), methicillin-resistant *Staphylococcus aureus* (MRSA), and methicillin-resistant *Staphylococcus epidermidis* (MRSE).

### Outcome measures

(1) Baseline data: Patient demographic and clinical data were collected, including age, body mass index (BMI), sex, and comorbidities such as hypertension, diabetes, and coronary heart disease (CHD).

(2) ICU-related information: ICU-related data included MDROs colonization status, length of ICU stay (prior to infection), APACHE and Chronic Health Evaluation II score within 24 h of ICU admission, use of central venous catheters (CVCs), ventilators, and urinary catheters, as well as the types and duration of antimicrobial agent use and corticosteroid therapy.

(3) Laboratory tests: Within 48 h of ICU admission, 3 mL of fasting venous blood was collected from each patient. Albumin (Alb) and fasting plasma glucose (FPG) levels were measured using a fully automated biochemical analyzer (URIT-8020A; Guilin Urit Medical Electronics Co., Ltd., Guilin Medical Device Registration No. 20172220142). White blood cell (WBC) count and hemoglobin (Hb) levels were measured using a fully automated hematology analyzer (XS-500ix, Jinan Xisen Meikang Medical Electronics Co., Ltd., Shandong Medical Device Registration No. 20182220002).

### Sample size calculation

According to previous studies, the incidence of MDROs infections among ICU patients ranges from 8.81 to 16.7% (1). The median incidence rate of 13.76% was used for the sample size estimation. This study included four variables in the logistic regression model. The sample size was calculated based on the events per variable (EPV) principle, with an EPV of 10. According to the formula, *n* = 4 × 10/13.76% ≈ 291 cases. Considering a potential 20% missing data rate, at least 364 patients were required for the study. Ultimately, 388 patients who met the inclusion and exclusion criteria were enrolled, satisfying the minimum sample size requirements.

### Statistical analysis

Data analysis was performed using SPSS version 27.0. Continuous variables with a normal distribution are expressed as mean ± standard deviation (*x̄* ± SD), and comparisons between groups were performed using the independent samples *t*-test. Categorical variables are expressed as frequencies and percentages [*n* (%)], and comparisons between groups were conducted using the chi-square test.

Using R version 4.4.3, 70% of the data were randomly assigned to the training set, and the remaining 30% were assigned to the validation set. Based on clinical observations (independently assessed by clinicians; in cases of disagreement, consensus was reached through discussion or consultation with a third senior physician) and variables with *p* < 0.05 from the aforementioned tests, binary logistic regression analysis was performed on the training set to identify independent factors associated with MDROs infection in ICU patients. Based on these results, a nomogram prediction model was constructed. The predictive performance of the model was evaluated using the area under the curve (AUC), calibration, and receiver operating characteristic (ROC) curves. Statistical significance was set at *p* < 0.05.

## Results

### Colonization and distribution of MDROs in ICU patients

Among the 388 ICU patients, 99 were colonized by MDROs, resulting in a colonization rate of 25.52% (95%CI: 21.44–30.08%). A total of 108 MDROs strains were detected, including 29 strains of ESBL-EC (26.61%), 54 strains of ESBL-KP (49.54%), 16 strains of MRSA (14.68%), and 9 strains of MRSE (9.17%).

### Baseline comparability between the training and validation sets

Comparisons between the training and validation sets showed no statistically significant differences in baseline characteristics or clinical variables (all *p* > 0.05), indicating good comparability between the two datasets and supporting the feasibility of the internal validation.

### Univariate analysis of MDROs infections in ICU patients in the training set

Comparison of clinical data between the two groups showed that patients in the infection group had significantly longer ICU stays (*t* = −2.394, *p* = 0.017), higher APACHE II scores (*t* = −9.518, *p* < 0.001), and higher WBC counts (*t* = −2.128, *p* = 0.034) than those in the non-infection group. In contrast, Alb levels were significantly lower in the infection group than in the non-infection group (*t* = 7.274, *p* < 0.001). Significant differences were observed between the two groups with respect to diabetes (*χ*^2^ = 8.576, *p* = 0.003), MDROs colonization (*χ*^2^ = 33.875, *p* < 0.001), and glucocorticoid use (*χ*^2^ = 9.644, *p* = 0.002). Details are shown in Table 1.

**Table 1:** Univariate analysis of MDROs infections among ICU patients in the training set [*x̄* ± SD, *n* (%)].

Variables	Non-infection group (*n* = 240)	Infection group (*n* = 31)	*t/x^2^*	*P*
Age (years)	68.10 ± 8.63	68.07 ± 10.39	0.021	0.983
BMI (kg/m^2^)	21.14 ± 1.42	21.25 ± 0.96	−0.554	0.582
Sex			0.090	0.764
Male	123 (51.25)	15 (48.39)		
Female	117 (48.75)	16 (51.61)		
Hypertension			0.066	0.798
No	118 (49.17)	16 (51.61)		
Yes	122 (50.83)	15 (48.39)		
Diabetes			8.576	0.003
No	129 (53.75)	8 (25.81)		
Yes	111 (46.25)	23 (74.19)		
CHD			0.257	0.612
No	120 (50.00)	17 (54.84)		
Yes	120 (50.00)	14 (45.16)		
MDROs colonization			33.875	<0.001
No	193 (80.42)	10 (32.26)		
Yes	47 (19.58)	21 (67.74)		
ICU Length of Stay	9.00(6.00,12.00)	11.00(9.00,13.50)	−2.419	0.016
APACHE II Score	23.20 ± 1.51	26.03 ± 1.89	−9.518	<0.001
CVC			1.930	0.165
No	131 (54.58)	21 (67.74)		
Yes	109 (45.42)	10 (32.26)		
Ventilator			0.813	0.367
No	111 (46.25)	17 (54.84)		
Yes	129 (53.75)	14 (45.16)		
Urethral Catheter			0.303	0.582
No	119 (49.58)	17 (54.84)		
Yes	121 (50.42)	14 (45.16)		
Categories of Antibiotics (Types)			0.269	0.604
<3	112 (46.67)	16 (51.61)		
≥3	128 (53.33)	15 (48.39)		
Duration of Antimicrobial Use (d)	10.37 ± 2.32	10.03 ± 2.29	0.766	0.444
Glucocorticoid			9.644	0.002
No	133 (55.42)	8 (25.81)		
Yes	107 (44.58)	23 (74.19)		
WBC (×10^9^/L)	11.65 ± 3.07	12.92 ± 3.58	−2.128	0.034
Hb (g/L)	105.99 ± 6.26	104.61 ± 5.28	1.173	0.242
FPG (mmol/L)	7.23 ± 1.12	7.29 ± 1.04	−0.300	0.764
Alb (g/L)	35.64 ± 2.60	31.97 ± 2.96	7.274	<0.001

CHD, coronary heart disease; CVC, central venous catheter.

### Logistic regression analysis of MDROs infections in ICU patients in the training set

Variables with *p* < 0.05 in the univariate analysis of the training set were included in the binary logistic regression analysis (Table 2). The occurrence of MDROs infection in ICU patients was used as the dependent variable (1 = infection, 0 = no infection). The independent variables included diabetes (1 = yes, 0 = no), MDROs colonization (1 = yes, 0 = no), ICU length of stay (original value), APACHE II score (original value), glucocorticoid use (1 = yes, 0 = no), WBC count (original value), and Alb level (original value).

**Table 2:** Logistic regression analysis of MDROs infections in ICU Patients.

	*B*	SE	Wald	*P*	OR	95%CI
Diabetes	0.926	0.426	4.724	0.030	2.523	1.095–5.814
MDROs Colonization	1.640	0.411	15.892	<0.001	5.155	2.302–11.544
ICU Length of Stay	0.089	0.054	2.687	0.101	1.093	0.983–1.215
APACHE II	0.715	0.126	32.284	<0.001	2.043	1.597–2.614
Glucocorticoid	0.781	0.417	3.502	0.061	2.184	0.964–4.948
WBC	0.067	0.063	1.132	0.287	1.070	0.945–1.211
Alb	−0.250	0.070	12.793	<0.001	0.778	0.679–0.893
Constant	−14.049	4.039	12.099			

The results showed that diabetes (OR = 2.523, 95% CI: 1.095–5.814), MDROs colonization (OR = 5.155, 95% CI: 2.302–11.544), APACHE II score (OR = 2.043, 95% CI: 1.597–2.614), and Alb level (OR = 0.778, 95% CI: 0.679–0.893) were independent factors associated with MDROs infection in ICU patients.

The logistic regression equation was as follows: *p* = −14.049 + 0.926 × diabetes + 1.640 × MDROs Colonization + 0.089 × ICU Length of Stay + 0.715 × APACHE II Score + 0.781 × Glucocorticoid Use + 0.067 × WBC − 0.250 × Alb.

### Logistic regression nomogram for MDROs infection in ICU patients

**Figure 1:**
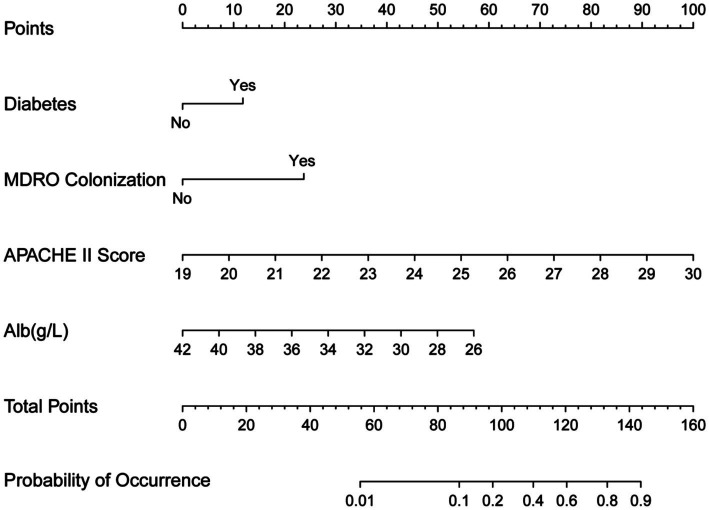
Nomogram model for predicting MDROs infections in ICU Patients.

Variables with *p* < 0.05 in the multivariate logistic regression analysis were included in the construction of the nomogram (Figure 1). Each risk factor was assigned a corresponding score, and the probability of MDROs infection in ICU patients was estimated according to the total score obtained by summing the scores of all variables.

**Figure 2:**
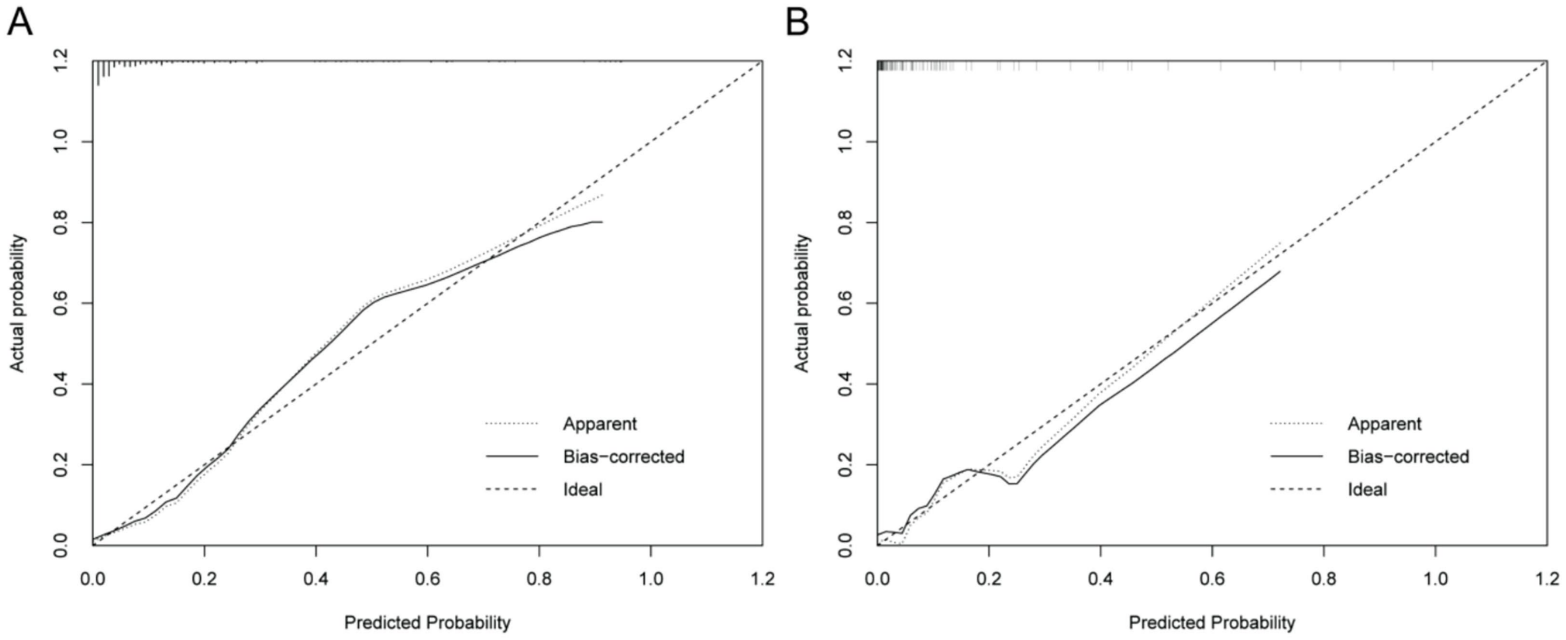
Calibration curves for the nomogram model. **(A)** Training set; **(B)** validation set.

The results indicated that diabetes, MDROs colonization, high APACHE II scores, and low Alb levels were significant factors associated with MDROs infection in ICU patients. ROC curves for the nomogram were generated for both the training and validation sets (Figures 2A,B). The mean absolute errors were 0.0009 and 0.0033, respectively. The results demonstrated good predictive performance and agreement between the predicted probabilities and observed outcomes.

### ROC curve

**Figure 3:**
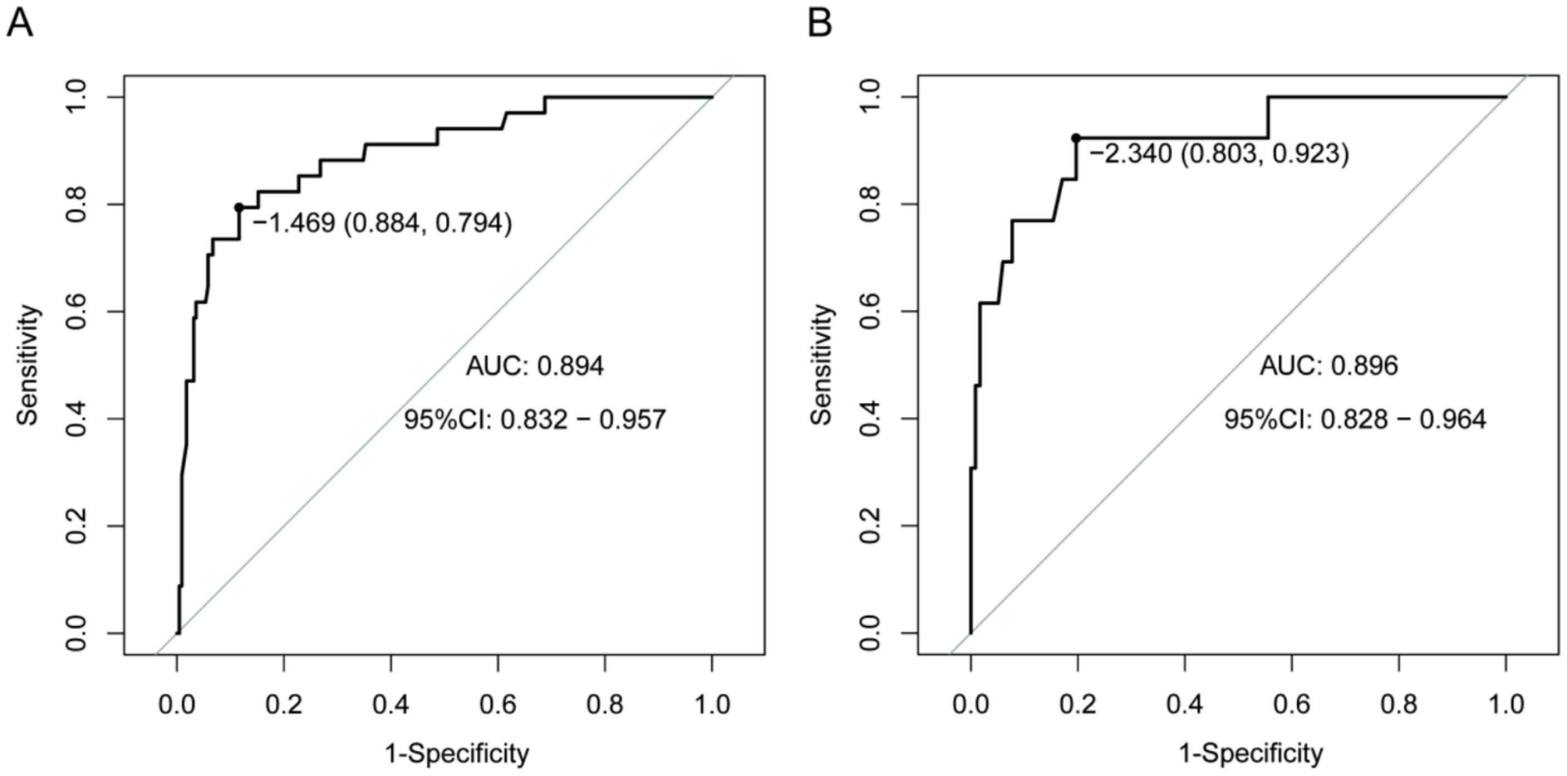
ROC curve. **(A)** Training set; **(B)** validation set.

The ROC curves were generated to evaluate the predictive performance of the nomogram model for MDROs infection in ICU patients. The results showed that AUC values for the training and validation sets were 0.894 (95% CI: 0.832–0.957) and 0.896 (95% CI: 0.828–0.964), respectively (Figures 3A,B).

## Discussion

The ICU is a specialized hospital unit dedicated to the centralized management of critically ill patients. These patients typically have severe underlying diseases and acute physiological dysfunction and are often immunocompromised. Combined with the frequent use of invasive procedures in the ICU, these factors make the ICU a high-risk setting for infections caused by MDROs (9). This study found that the colonization rate of MDROs among ICU patients was 25.52%, which was slightly higher than that reported in previous studies (10). A total of 108 MDROs strains were detected, among which ESBL-KP accounted for the highest proportion (49.54%). This may be attributed to the strong plasmid transmission ability and clonal dissemination potential of ESBL-KP, which facilitates cross-transmission among ICU patients (11, 12). In contrast, the colonization rates of MRSA and MRSE were relatively low, possibly due to the infection control measures targeting gram-positive cocci implemented in recent years (13, 14). These findings suggest that infection control strategies in ICUs should prioritize active screening and isolation of ESBL-producing Enterobacteriaceae, particularly ESBL-producing *K. pneumoniae*.

This study identified diabetes mellitus as an independent risk factor for MDROs infection in patients in the ICU. A possible explanation is that chronic hyperglycemia impairs the chemotaxis, phagocytosis, and bactericidal functions of neutrophils, while also compromising the integrity of the skin and mucosal barriers (15, 16). In addition, patients with diabetes often experience microvascular complications and tissue hypoxia, which may increase the risk of infection following MDROs colonization (17). MDROs colonization was also identified as a risk factor for infection in ICU patients, consistent with previous findings (18). This may be because invasive procedures, such as central venous catheterization, mechanical ventilation, and urinary catheterization, can disrupt host defense barriers and facilitate the entry of colonizing MDROs from the skin or mucosal surfaces into the body (19, 20). Furthermore, impaired immune function in ICU patients may promote the translocation of locally colonized MDROs to the bloodstream. Previous studies have demonstrated that patients colonized with MDROs have a significantly increased risk of subsequent infection caused by the same bacterial strain and that there is often anatomical continuity between colonization and infection sites, such as respiratory tract colonization progressing to ventilator-associated pneumonia (21). Therefore, enhanced infection control measures, including contact isolation and appropriate antibiotic stewardship, should be implemented for patients identified through active screening to interrupt the progression from colonization to infection (22, 23).

In addition, this study demonstrated that high APACHE II scores and low Alb levels were independent factors associated with MDROs infection in ICU patients. The APACHE II score is widely used to assess disease severity in ICU patients, and higher scores often reflect conditions such as multiple organ dysfunction or shock, which may increase susceptibility to MDROs infection (24). Low Alb levels may indicate poor nutritional status and systemic inflammation. Albumin serves as an important nutritional indicator and contributes to endotoxin binding and transport, as well as maintenance of plasma colloid osmotic pressure (25). In patients with low Alb levels, alterations in antibiotic pharmacokinetics, such as increased volume of distribution and reduced free drug concentration, may impair bacterial clearance and facilitate persistent colonization (26). The nomogram constructed based on these variables demonstrated good predictive performance, with AUC values of 0.894 and 0.896 in the training and validation sets, respectively. These findings suggest that the model may help clinicians identify high-risk patients at an early stage and optimize prevention and treatment strategies, including isolation intensity, antibiotic selection, and frequency of active monitoring.

In summary, the colonization rate of MDROs among ICU patients was 25.52%, with ESBL-KP identified as the predominant colonizing bacterium. Diabetes, MDROs colonization, high APACHE II scores, and low Alb levels were identified as independent factors associated with MDROs infection in ICU patients. The nomogram developed in this study demonstrated good predictive performance and may provide valuable support for clinical decision making. However, this study has several limitations. As a single-center retrospective study with a relatively small sample size, potential confounding factors, such as cross-infection and antibiotic dosage, may not have been fully accounted for, potentially introducing selection and outcome biases. Future multicenter prospective studies with larger sample sizes are warranted to standardize the active screening protocols. In addition, integrating genetic sequencing and additional clinical indicators may further clarify the relationship between colonizing and pathogenic bacteria, improve the predictive accuracy of MDROs infection models, and evaluate whether model-based stratified interventions can reduce the incidence of MDROs infections and improve patient outcomes.

## Conclusion

Diabetes, MDROs colonization, high APACHE II scores, and low albumin levels were independent factors associated with MDROs infection in ICU patients. The nomogram developed in this study demonstrated good predictive performance and may facilitate the early identification of patients at high risk of MDROs infection, thereby supporting the development of individualized prevention and treatment strategies.

## Data Availability

The original contributions presented in the study are included in the article/supplementary material, further inquiries can be directed to the corresponding author.
